# Giant Anterior Mediastinal Hamartoma in a Middle-Aged Woman: A Rare Encounter

**DOI:** 10.7759/cureus.103558

**Published:** 2026-02-13

**Authors:** Akanksha Soni, Fatima Inam, Sai Ram Kumar Yatiraju, Basma Alleelwa

**Affiliations:** 1 Acute Medical Unit, Stockport NHS Foundation Trust, Stockport, GBR; 2 Respiratory Medicine Department, Stockport NHS Foundation Trust, Stockport, GBR

**Keywords:** anterior mediastinum, hamartoma, mediastinal mass, popcorn calcification, thoracic radiology

## Abstract

Hamartomas are benign neoplasms most commonly arising in the lung, typically identified as incidental solitary pulmonary nodules. Occurrence in the mediastinum is exceedingly rare. Because anterior mediastinal lesions are most often malignant, hamartomas in this location pose a diagnostic challenge.

We present the case of a 53-year-old female with a background of endometrial carcinoma who was evaluated for chest pain and palpitations. A chest radiograph revealed a large opacity in the left upper hemithorax, corresponding to a well-circumscribed anterior mediastinal mass on computed tomography. The lesion measured 7.7 × 7 × 7 cm, contained macroscopic fat, and partially compressed adjacent bronchovascular structures. A CT-guided biopsy demonstrated fibrous, cartilaginous, adipose, and bronchial elements without atypia, confirming the diagnosis of mediastinal hamartoma. Due to the lesion’s size and compressive effects, surgical excision was performed through a left exploratory thoracotomy. The patient recovered well postoperatively.

Mediastinal hamartomas are rare and can mimic more aggressive anterior mediastinal neoplasms. Imaging features such as fat density may suggest the diagnosis, but histopathology remains essential for confirmation. Surgical resection is indicated in symptomatic or compressive cases, and the prognosis following excision is excellent. This case highlights the importance of considering benign entities such as hamartoma in the differential diagnosis of anterior mediastinal masses.

## Introduction

Hamartomas are benign tumors with disorganized but mature mesenchymal and epithelial components typical of their tissue of origin. They can arise in any organ, but appear most often in the lung, where they account for 5-8% of solitary pulmonary nodules and up to 75% of benign pulmonary tumors. Pulmonary hamartomas are common, but mediastinal hamartomas are extremely rare, with few documented cases in the English literature [1,2]. Most anterior mediastinal lesions are malignant, so diagnosing hamartomas in this location is challenging. We present the case of a 53-year-old female and discuss her diagnosis and management.

## Case presentation

**Figure 1:**
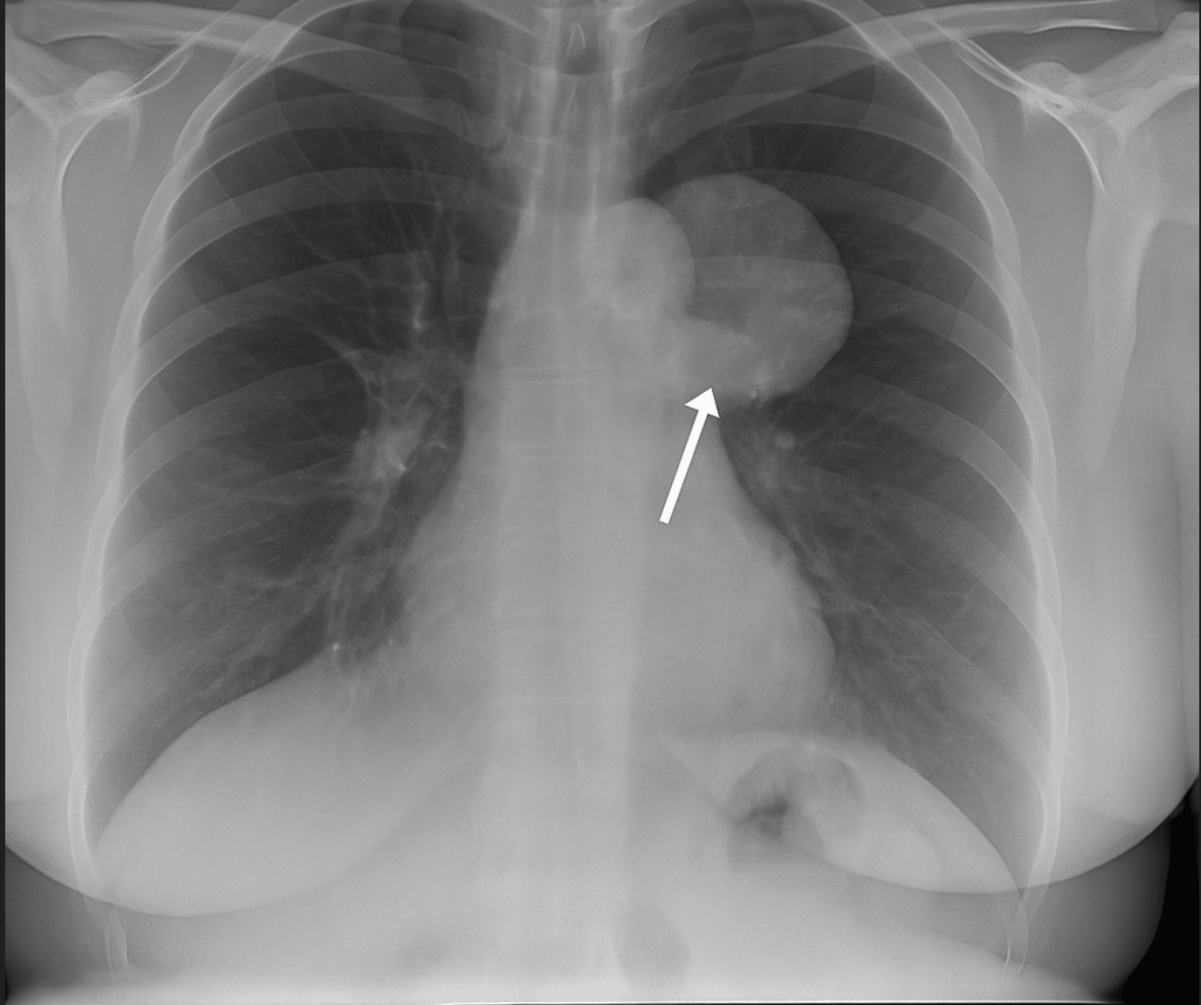
Chest radiograph (posteroanterior view) showing a well-defined, rounded opacity in the left upper mediastinal region.

A 53-year-old female presented with chest pain and palpitations at Medical Same Day Emergency Care. She had a background of endometrial carcinoma. A routine chest radiograph incidentally revealed a left-sided mediastinal mass. The lesion was first identified in 2017, but remained asymptomatic and was not biopsied at that time. The patient remained clinically well until 2025, when her new symptoms prompted further imaging. Given her chest X-ray findings (Figure 1), an outpatient CT thorax and chest clinic appointment was arranged for her.

**Figure 2:**
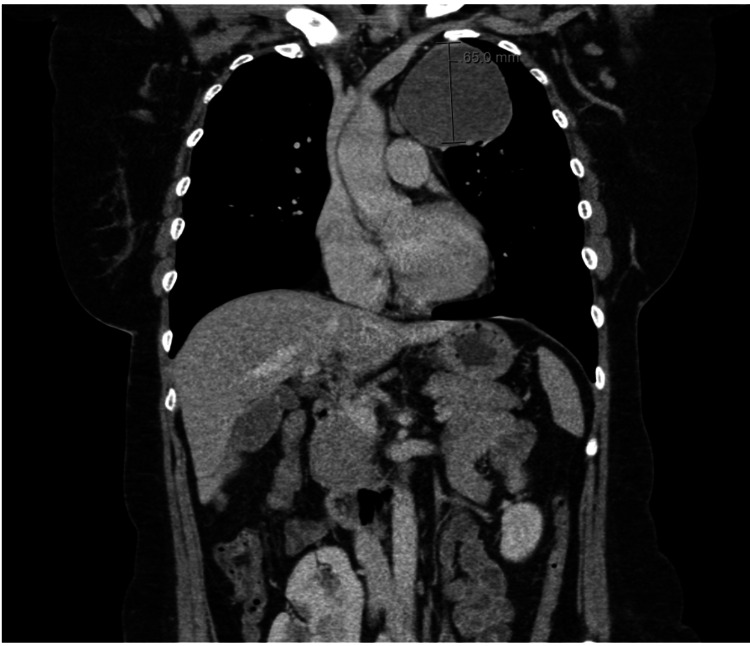
Coronal view contrast-enhanced CT thorax showing a well-defined anterior mediastinal hamartoma

**Figure 3:**
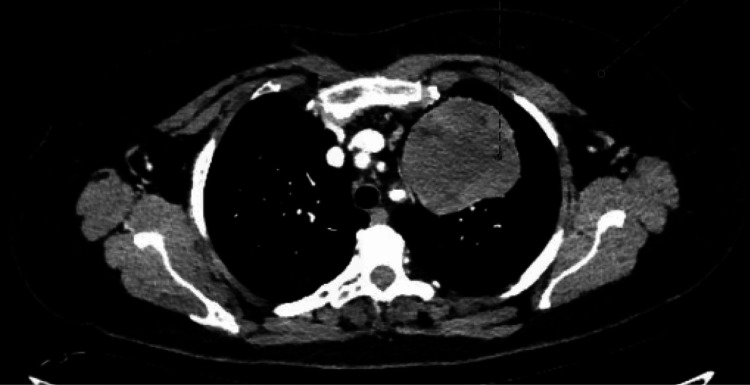
Axial view contrast-enhanced CT thorax showing a well-circumscribed anterior mediastinal hamartoma with heterogeneous attenuation, containing focal areas of macroscopic fat, causing mild mass effect on adjacent mediastinal structures without invasion.

A contrast-enhanced CT scan of the thorax demonstrated a large, well-defined mass (7.7 × 7 × 7 cm) situated in the medial aspect of the upper left hemithorax, contiguous with the anterior mediastinal fat (Figures 2-3). The lesion exhibited heterogeneous attenuation with areas of macroscopic fat and displaced adjacent airway and vascular structures. Importantly, no calcification, fluid content, or significant lymphadenopathy was seen.

**Figure 4:**
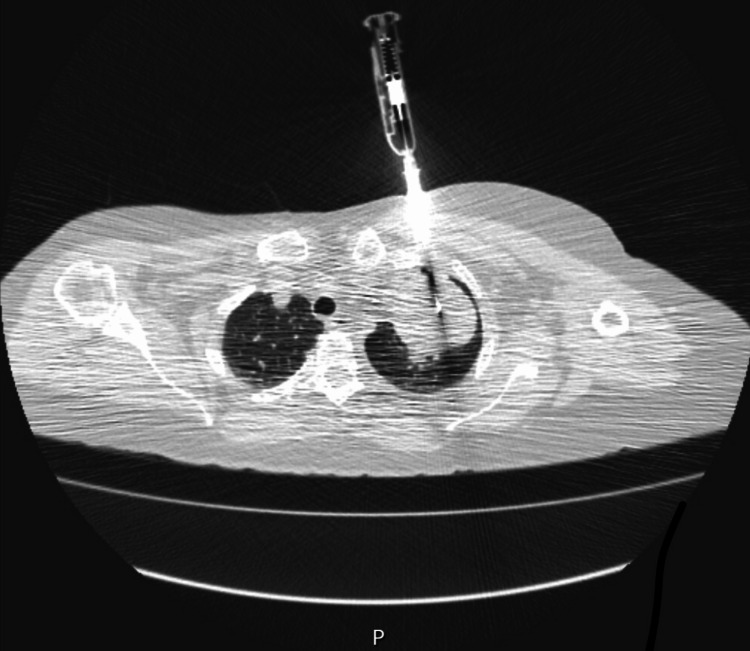
CT-guided core needle biopsy of the anterior mediastinal mass demonstrating accurate needle placement within the lesion for histopathological confirmation of hamartoma.

**Figure 5:**
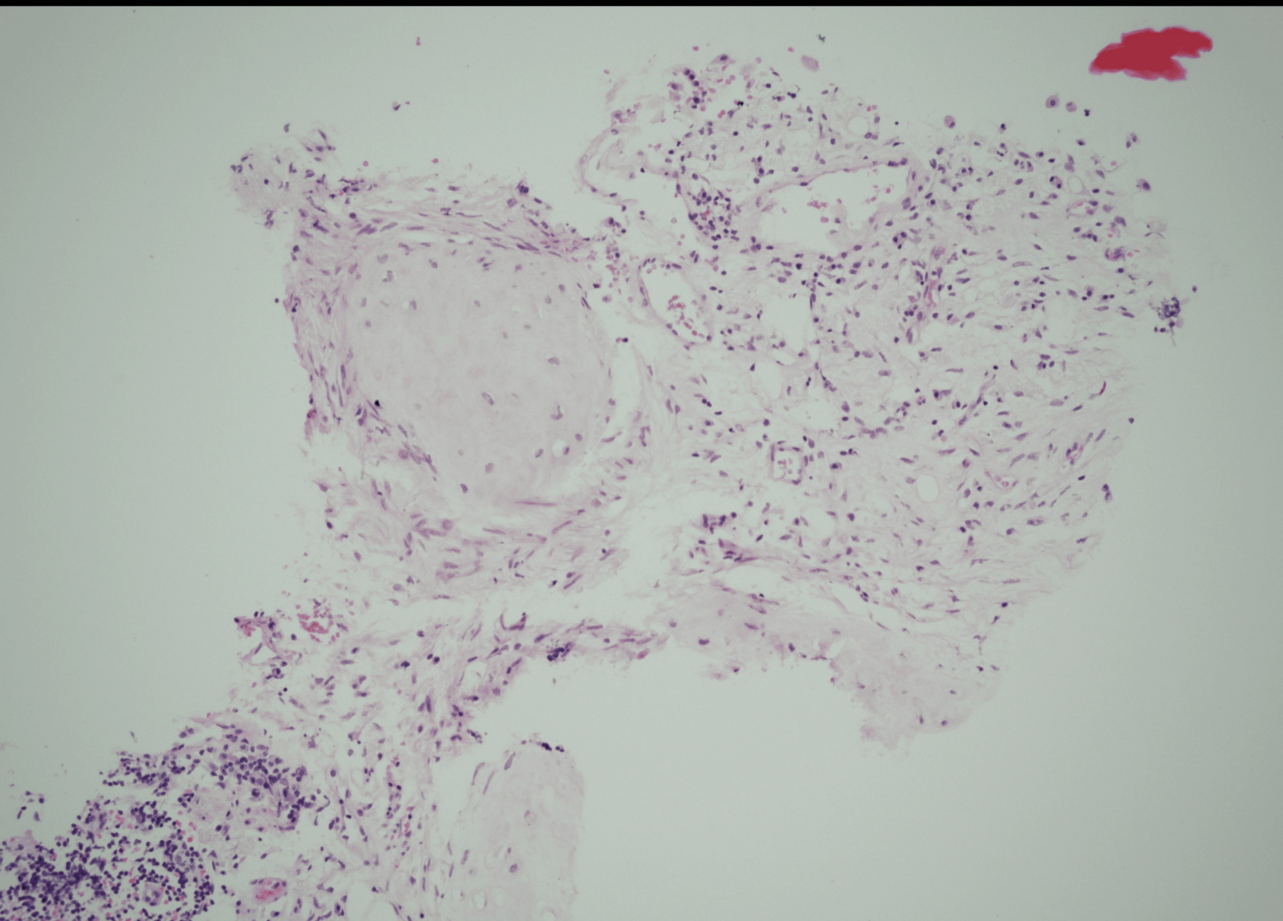
Histopathological section (H&E stain) showing disorganised fibromyxoid tissue containing cartilage, smooth muscle, and adipose elements consistent with hamartoma.

A CT-guided percutaneous biopsy was performed (Figure 4). Histopathology revealed a combination of bronchial mucosa, myxoid fibrous tissue, mature adipose tissue, smooth muscle, and cartilaginous elements, with no features of malignancy (Figure 5). These findings were diagnostic of a mediastinal hamartoma. The absence of immature tissue, thymic remnants, or atypia helped rule out teratoma, thymic neoplasms, and other malignant differentials.

Although benign, the size and mass effect of the lesion, including partial compression of the left upper lobe bronchus and encasement of the segmental pulmonary artery, posed a potential risk for future complications. Based on these factors and following the discussion at the multidisciplinary team (MDT) meeting, surgical excision was advised. The patient consented to a left exploratory thoracotomy with resection of the mass. Her excellent pulmonary function tests (PFTs) and good functional status contributed to her being an excellent candidate for surgery.

## Discussion

Histologically, hamartomas typically contain at least two mesenchymal elements, such as cartilage and fat, intermixed with fibrous tissue, smooth muscle, and bronchial epithelium [1,2]. They generally occur in middle-aged individuals and are often incidentally detected during imaging for unrelated conditions [1,2]. Because mediastinal masses are more frequently malignant - most commonly thymomas and lymphomas in the anterior mediastinum - hamartomas in this region present a significant diagnostic challenge [3]. Radiologically, mediastinal hamartomas can resemble more aggressive diseases. Chest X-rays are often nonspecific. Computed tomography (CT) scans may reveal a well-defined mass with fat density and occasional calcification [4,5]. However, differentiating a benign hamartoma from a malignant tumor frequently requires histopathological confirmation [1,2]. Due to their rarity and nonspecific presentation, mediastinal hamartomas are often diagnosed incidentally. Most cases are surgically excised because of diagnostic uncertainty, compressive symptoms, or progressive growth [1,2,3].

A hamartoma is a developmental anomaly composed of an excessive proliferation of mature tissue native to the site of origin. This tissue is arranged in an abnormal architectural pattern [6]. Pulmonary hamartomas account for 5-8% of solitary pulmonary nodules and nearly 75% of benign pulmonary tumors [2,4]. They are approximately four times more common in men than in women in peripheral parenchymal locations. In contrast, central lesions near the hilar region show equal sex distribution [7]. Most patients present between the fourth and seventh decades of life [7]. Histologically, these tumors are characterized by mesenchymal components-most commonly cartilage and fat-combined with fibrous or smooth muscle tissue and bronchial epithelium [1,2].

In contrast, mediastinal hamartomas are exceedingly rare. Only a few cases are documented in the literature [1,3]. The anterior mediastinum is predominantly the site of malignant tumors, particularly thymomas and lymphomas, which together constitute over 60% of masses in this region [3,7]. Consequently, mediastinal hamartomas can be difficult to distinguish radiologically from malignant lesions.

Radiographic findings of mediastinal hamartomas are nonspecific. On chest radiography, they appear as well-circumscribed opacities. CT imaging typically reveals a heterogeneous mass with areas of fat attenuation and, occasionally, calcification. In our patient, CT demonstrated a large, well-circumscribed anterior mediastinal mass containing macroscopic fat but lacking calcification. This further emphasizes the diagnostic overlap with malignant neoplasms.

Histopathological examination remains the gold standard for diagnosis. In this case, CT-guided biopsy demonstrated fibrous tissue with myxoid areas, mature adipocytes, smooth muscle, cartilaginous components, and bronchial epithelium. These findings are consistent with previously reported hamartomas [1,2,3]. The absence of atypia, immature tissue, or thymic remnants helped exclude differentials such as teratoma, thymoma, and other malignant tumors.

Management strategies for mediastinal hamartomas depend on lesion size, symptoms, and diagnostic confidence. Small, asymptomatic lesions may be observed with serial imaging. However, large or symptomatic lesions typically warrant surgical excision [1,4]. In our case, resection was advised due to the lesion’s size and its compression of bronchovascular structures. Surgical excision is considered curative. Recurrence is exceptionally rare [1,5].

This case highlights three key points. First, anterior mediastinal hamartomas, though rare, should be included in the differential diagnosis of anterior mediastinal masses when imaging demonstrates fat-containing lesions. Second, long-term imaging stability supports a benign nature but does not eliminate the need for histological confirmation. Third, surgical excision remains the definitive management for symptomatic or diagnostically uncertain lesions. It offers both curative and diagnostic benefits.

## Conclusions

Mediastinal hamartomas are a rare subset of benign thoracic tumors, with very few cases described in the literature. Their presentation may mimic malignant anterior mediastinal lesions such as thymoma, lymphoma, or germ cell tumors, leading to diagnostic uncertainty. Imaging findings of a well-circumscribed lesion with areas of fat are suggestive but not definitive, as classic features such as “popcorn” calcification are not always present.

Histopathology remains essential for definitive diagnosis, as demonstrated in this case and others. Although benign, surgical resection is often recommended when the lesion is large, symptomatic, or causing compression of adjacent structures, and the prognosis following complete excision is excellent. This case adds to the small body of literature on mediastinal hamartomas and highlights the need to consider this rare benign entity in the differential diagnosis of anterior mediastinal masses.
